# Stage 1 of the meaningful use incentive program for electronic health records: a study of readiness for change in ambulatory practice settings in one integrated delivery system

**DOI:** 10.1186/s12911-014-0119-1

**Published:** 2014-12-14

**Authors:** Christopher M Shea, Kristin L Reiter, Mark A Weaver, Molly McIntyre, Jason Mose, Jonathan Thornhill, Robb Malone, Bryan J Weiner

**Affiliations:** Department of Health Policy and Management, University of North Carolina-Chapel Hill, Chapel Hill, NC USA; Cecil G. Sheps Center for Health Services Research, University of North Carolina-Chapel Hill, Chapel Hill, NC USA; School of Medicine and Department of Biostatistics, University of North Carolina-Chapel Hill, Chapel Hill, NC USA; CareFirst BlueCross BlueShield, Baltimore, MD USA; UNC Faculty Physicians, Department of Practice Quality and Innovation, UNC Health Care, Chapel Hill, NC USA; Lineberger Comprehensive Cancer Center, University of North Carolina-Chapel Hill, Chapel Hill, NC USA

## Abstract

**Background:**

Meaningful Use (MU) provides financial incentives for electronic health record (EHR) implementation. EHR implementation holds promise for improving healthcare delivery, but also requires substantial changes for providers and staff. Establishing readiness for these changes may be important for realizing potential EHR benefits. Our study assesses whether provider/staff perceptions about the appropriateness of MU and their departments’ ability to support MU-related changes are associated with their reported readiness for MU-related changes.

**Methods:**

We surveyed providers and staff representing 47 ambulatory practices within an integrated delivery system. We assessed whether respondent’s role and practice-setting type (primary versus specialty care) were associated with reported readiness for MU (i.e., willingness to change practice behavior and ability to document actions for MU) and hypothesized predictors of readiness (i.e., perceived appropriateness of MU and department support for MU). We then assessed associations between reported readiness and the hypothesized predictors of readiness.

**Results:**

In total, 400 providers/staff responded (response rate approximately 25%). Individuals working in specialty settings were more likely to report that MU will divert attention from other patient-care priorities (12.6% vs. 4.4%, p = 0.019), as compared to those in primary-care settings. As compared to advanced-practice providers and nursing staff, physicians were less likely to have strong confidence in their department’s ability to solve MU implementation problems (28.4% vs. 47.1% vs. 42.6%, p = 0.023) and to report strong willingness to change their work practices for MU (57.9% vs. 83.3% vs. 82.0%, p < 0.001). Finally, provider/staff perceptions about whether MU aligns with departmental goals (OR = 3.99, 95% confidence interval (CI) = 2.13 to 7.48); MU will divert attention from other patient-care priorities (OR = 2.26, 95% CI = 1.26 to 4.06); their department will support MU-related change efforts (OR = 3.99, 95% CI = 2.13 to 7.48); and their department will be able to solve MU implementation problems (OR = 2.26, 95% CI = 1.26 to 4.06) were associated with their willingness to change practice behavior for MU.

**Conclusions:**

Organizational leaders should gauge provider/staff perceptions about appropriateness and management support of MU-related change, as these perceptions might be related to subsequent implementation.

## Background

The Centers for Medicare and Medicaid Services Meaningful Use (MU) program provides financial incentives to hospitals and eligible providers for adopting a certified electronic health record (EHR). As of June 2014, the program had disbursed more than $24 billion in incentive payments [1]. Underlying this large investment in EHR adoption across the health care system is the belief that EHRs are better tools for supporting efficient, high quality care than are paper records [2]. However, simply purchasing an EHR does not ensure delivery of more efficient and higher quality care; the EHR must also be used effectively. Therefore, to receive all available financial incentives through the MU program, providers must demonstrate “meaningful use” by meeting specific MU objectives that cover a range of clinical processes (e.g., documenting vital signs and problems, ordering lab tests and medications, and providing visit summaries and educational materials to patients) [3]. Changing these processes within a practice setting to demonstrate MU requires a coordinated effort by providers and clinical staff [4].

In busy practice settings, such change efforts are often difficult to implement effectively. In fact, experts have suggested that without sufficient readiness for change, change efforts are more likely to lead to unrealized benefits or fail altogether [5,6]. With billions of dollars invested in MU and the countless hours spent by providers and clinical staff on MU implementation nationally, unrealized benefits from the program would carry significant financial and opportunity costs for health care systems. Therefore, understanding readiness for change in the context of MU is important for leaders at both the healthcare-organization level and the state and national policy levels. Despite the importance of effective implementation of MU, however, there has been little empirical research on assessing readiness for change to demonstrate MU.

Although research on readiness for change is still evolving, there has been foundational work to synthesize the literature [7,8], develop theory [9], and develop measurement tools [10,11] for assessing readiness for change in health care settings. A small number of studies have focused on readiness of physicians, nurses, and other providers for organizational changes that are not specific to information technology (IT) [12]. However, the only article we identified that focused on readiness for health-IT related change reported mixed evidence from two studies—one of home care organizations and one of a large teaching hospital. For example, the presence of an effective champion and top management support each were statistically significant predictors in only one of the two studies [13]. Such results suggest that predictors of readiness may be context dependent, with some variables being important for only some changes in some organizations. We could not locate any studies that focused on readiness for change in the context of MU.

Although we are unaware of previous studies that have examined associations between readiness for MU and individual or practice-setting characteristics, evidence suggests associations between EHR adoption and practice-setting characteristics, such as number of providers and number of specialties represented, as well as physician characteristics, such as primary care provider vs. specialist [14,15]. Also, there is evidence that primary care providers view some MU objectives as being more important than do specialists [16], a belief that could influence perceptions about appropriateness, support, and readiness related to MU implementation. Furthermore, evidence suggests that EHR implementations typically lead to at least temporary increases in workload [17] within the practice; however, it is unclear whether perceptions of the burden vary by role (e.g., physician or nurse).

The overall purpose of our study was to examine perceptions among health care providers and staff about the MU program and their readiness for implementing it. First, we explored whether individuals’ perceptions about the appropriateness of MU, perceived departmental support for MU, and perceived readiness for MU were associated with their practice type (i.e., primary care vs. specialty) or their roles within the practice (i.e., physicians, advanced practice providers, and nursing staff). Based on the available literature, we expected that individuals in primary care settings would report higher perceived appropriateness of MU, greater departmental support for MU, and higher readiness for MU than would individuals in specialty settings. We did not formulate any expectations related to individuals’ roles in their clinical settings.

Second, we examined whether provider and staff perceptions about the appropriateness of MU and their department’s ability to support their MU-related change efforts were associated with their perceived readiness to implement MU-related change. Literature from the field of implementation science suggests that readiness for change involves both psychological and behavioral components—that is, both willingness and ability to make a specific change [7,9]. Furthermore, perceived appropriateness of the impending change and management support are commonly cited predictors of readiness for change [10,11,13,18]. We were interested in two aspects of appropriateness of the change—(1) the fit between MU and the department’s goals for patient care and (2) the extent to which MU would divert attention from other high priority patient care activities. We were also interested in two aspects of management support—(1) overall support for MU effort and (2) ability to solve problems that would hinder productivity during MU implementation. We expected that these variables would be positively associated with both individuals’ willingness to change for MU and perceived ability to demonstrate MU. It is important to note that our study design did not allow for testing a predictive model.

## Methods

### Study setting

Our study included ambulatory practice settings within University of North Carolina Health Care System (UNC HCS). UNC HCS is a not-for-profit integrated health care system owned by the state of North Carolina. All clinics in the study were using the same EHR and operating under the same system-level policies for MU implementation, including how incentive payments were distributed. In addition, each clinic had access to the same types of support (e.g., training, process improvement coaching) and contributed financially to cover the costs of this support.

### Sample

Using various UNC HCS listservs and messages distributed through the EHR, we recruited clinicians and clinical support staff to complete our MU readiness survey. Responses were collected in November and December of 2011, which was approximately three months after UNC HCS launched its campaign to communicate with providers and staff about the upcoming MU implementation but still prior to implementation process changes. We included in our sample all respondents who: (1) reported being physicians, advanced practice providers, or nursing staff, (2) indicated they use the EHR for clinical purposes (i.e., not exclusively for research or administrative purposes) and (3) have clinical patient contact.

### Measures

We identified no validated measures of readiness for change that would be practical for our study setting given UNC HCS leadership’s desire for a brief survey. Existing measures we identified included 40 or more items [10,11,13]. We therefore developed a brief web-based survey instrument that would measure our primary variables of interest and also collect information that would be useful for UNC HCS leaders. We pre-tested our survey on clinicians in UNC HCS. Responses obtained during pretesting were not included in our analytic dataset. (Note: Since the completion of our data collection, a briefer measure of organizational readiness for change has been published) [19].

The final version of the survey consisted of 14 items covering demographics (i.e., role, length of time in current role, primary care or specialty care setting, frequency of EHR use per week); readiness for MU change (i.e., willingness to change practice behavior for MU, ability to respond to MU-required quality prompts in the EHR); appropriateness of MU (i.e., perceived fit of MU with department goals, perceived fit of MU with patient care priorities); and management support for the MU effort (i.e., perceived departmental support for individuals pursuing MU, departmental ability to solve problems that arise during MU implementation). Our survey item on willingness to change practice behavior was based upon the psychological component of readiness (i.e., willingness) [7] and was of interest to UNC HCS system leadership. Our item about ability to document action in response to quality prompts (e.g., cancer screening, foot exam, labs) was similar to efficacy items in other instruments that refer to “confidence” in performing a task [10]. Although we asked about ability to perform other tasks as well, we focused our analysis on this particular indication because it is applicable for all the different roles included in our sample. Our items about the alignment of MU with departmental goals, patient care priorities, and overall departmental support for MU were similar to items used in other studies [10,13]. The item about the department’s ability to solve problems that would hinder productivity was developed based on the interests of UNC HCS leadership. All of these items used a four-point Likert scale (ranging from “not at all willing” to “very willing,” “not at all confident” to “very confident,” or “strongly disagree” to “strongly agree”), with two additional options for “Don’t Know” and “Not Applicable.”

### Analysis

First, we classified the practice settings as primary care (including family medicine, internal medicine, primary obstetrics/gynecology, geriatrics, and pediatrics) or specialty care (all others) and the respondents’ roles as physician (faculty/attending and resident/fellow), advanced practice (e.g., nurse practitioner, clinical social worker), or nurse. For all analyses, we dichotomized self-reported ability, self-reported willingness, and the four predictors of willingness so that the most positive response (i.e., “very confident”, “very willing”, or “strongly agree”) = 1 and other responses = 0. We fit all models using generalized estimating equation methods to account for clustering at the clinical division level. Responses of “don’t know” or “not applicable” were set to missing and not included in comparisons. To explore whether perceptions about the MU program and readiness for implementing MU were associated with either practice setting type or respondent’s role, we applied 12 separate logistic regression models, one for each characteristic-by-outcome combination (analogous to conducting separate chi-square tests, only adjusted for clustering). We then used separate multiple logistic regression, controlling for respondent’s role, to assess whether associations existed between respondents’ perceptions of the predictors of readiness and either their reported willingness or ability to demonstrate MU. We considered P < 0.05 to indicate statistically significant associations, with no adjustments for multiple comparisons.

All analyses were conducted using SAS, version 9.2 (SAS Institute, Cary, NC). This study was reviewed and received ethics approval and a waiver of written informed consent by the University of North Carolina’s Institutional Review Board (Study # 11-1032). Completion of the survey constituted implied consent.

## Results

### Characteristics of study sample

The final sample included 400 responses and represented clinical areas in 47 UNC HCS ambulatory care departments/divisions. Because our recruitment strategy included messages via listserv and the EHR, we were not able to identify the precise number of individuals who received the invitation to participate. However, using data from an inventory of roles collected during a separate study [20], we were able to estimate the number of eligible respondents to be 1,614 and, therefore, a response rate of approximately 25%. The highest percentages of the 400 total responses were from physicians (69.9%), and from individuals that had been in their role for 1-4 years (41.0%), practiced in specialty care clinics (65.5%), and used the EHR 5-7 days per week (86.7%). Table 1 provides more detail on respondent characteristics.

**Table 1:** **Summary of respondent characteristics (N = 400)**

**Characteristc**	**n (%)**
*Primary role*	
Physicians	280 (69.9)
Attending/faculty	159 (39.7)
Residents/fellows	121 (30.2)
Nurse	65 (16.2)
Advanced Practice Provider	55 (13.7)
*Type of care*	
Primary Care	140 (35.0)
Specialty Care	260 (65.0)
*Frequency of electronic health record use*	
Less than one day per week	2 (0.5)
One to two days per week	16 (4.0)
Three to four days per week	35 (8.7)
Five to seven days per week	347 (86.7)
*Length of time in role at institution*	
Less than one year	66 (16.5)
One to four years	164 (41.0)
Five to ten years	69 (17.2)
Over ten years	101 (25.2)

### Practice setting type and role

Our expectation related to practice setting was only partially supported (Table 2). Specifically, the belief that MU will divert attention from other important patient care activities was the only variable for which we observed a statistically significant association, with a higher percentage of individuals in specialty settings holding this belief than in primary care settings (12.6% vs. 4.4%, p = 0.019). Respondents’ willingness and ability to demonstrate MU were not significantly associated with their practice setting type.

**Table 2:** **Summaries of meaningful use readiness dimensions by respondents’ roles and practice setting type**

**Dimensions**	**Practice setting type**			**Role**			
	**Primary n (%)**	**Specialty n (%)**	P-value*	**Doctor n (%)**	**Nurse n (%)**	**Advanced practice provider n (%)**	P-value*
Individual Readiness							
Able to demonstrate			0.767				0.312
MU							
Very confident	34 (36.6)	63 (35.2)		62 (32.5)	21 (45.7)	14 (40.0)	
Less confident	59 (63.4)	116 (64.8)		129 (67.5)	25 (54.4)	21 (60.0)	
Missing	47	81		89	19	20	
Willing to change work practices for MU			0.932				<0.001
Very willing	82 (64.1)	155 (65.4)		151 (57.9)	45 (83.3)	41 (82.0)	
Less willing	46 (35.9)	82 (34.6)		110 (42.2)	9 (16.7)	9 (18.0)	
Missing	12	23		19	11	5	
Perceived departmental predictors							
Department will support MU demonstration			0.404				0.113
Strongly agrees	80 (64.0)	127 (55.2)		141 (55.5)	34 (61.8)	32 (69.6)	
Does not strongly agree	45 (36.0)	103 (44.8)		113 (44.5)	21 (38.2)	14 (30.4)	
Missing	15	30		26	10	9	
Department will solve problems to minimize productivity declines during MU implementation			0.393				0.023
Very confident	48 (38.4)	67 (30.0)		71 (28.4)	24 (47.1)	20 (42.6)	
Less confident	77 (61.6)	156 (70.0)		179 (71.6)	27 (52.9)	27 (57.5)	
Missing	15	37		30	14	8	
MU fits department's goals for patient care			0.786				0.375
Strongly agrees	62 (49.6)	83 (37.9)		100 (40.5)	24 (45.3)	21 (47.7)	
Does not strongly agree	63 (50.4)	136 (62.1)		147 (59.5)	29 (54.7)	23 (52.3)	
Missing	15	41		33	12	11	
MU diverts attention from other patient care activities			0.019				0.404
Strongly agrees	5 (4.4)	26 (12.6)		26 (10.8)	3 (7.5)	2 (5.0)	
Does not strongly agree	110(95.7)	180 (87.4)		215 (89.2)	37 (92.5)	38 (95.0)	
Missing	25	54		39	25	15	

*P-values for comparing across groups from generalized estimating equation model controlling for clustering by division.

Although we had no prior expectations about the respondent’s role, our exploratory analysis indicated a significant association with willingness to change work practices for MU, as physicians (57.9%) reported being less willing than advanced practice providers (82%) or nurses (83.3%) (p < 0.001). However, we did not identify a significant association between role and one’s perceived ability to demonstrate MU. Furthermore, physicians (28.4%) were significantly less likely to believe that their department would be able to address problems that arose during MU implementation as compared to advanced practice providers (42.6%) and nurses (47.1%) (p = 0.023). We also explored differences in responses between faculty/attending physicians and residents/fellows. We found faculty/attending physicians were less confident that their department would be able to address problems that arose during implementation than were residents/fellows; however, faculty/attending physicians were more willing to change their work practices for MU than were residents/fellows (data not shown).

### Precursors to readiness

Each of our measured predictors of readiness had a substantial and statistically significant association with respondents’ willingness to change their work practices for MU. The predictor with the largest observed association was the respondents’ belief that their department would support their efforts to demonstrate MU (OR = 3.99; 95% confidence interval = 2.13 to 7.48). However, none of the predictors had a statistically significant association with respondents’ ability to demonstrate MU (Table 3).

**Table 3:** Associations between hypothesized predictors of readiness and participants’ reported willingness and ability to demonstrate meaningful use*

**Perceived predictor**	**Willingness to change work practices for meaningful use**			**Ability to demonstrate meaningful use**		
	**OR**	**95% CI**	**P-value**	**OR**	**95% CI**	**P-value**
Department will support MU demonstration	3.99	(2.13, 7.48)	<0.001	1.23	(0.63, 2.37)	0.545
Department will solve problems to minimize productivity declines during MU implementation	2.26	(1.26, 4.06)	0.006	0.72	(0.34, 1.51)	0.384
MU fits department's goals for patient care	1.98	(1.27, 3.07)	0.002	0.60	(0.32, 1.14)	0.118
MU diverts attention from other patient care activities	0.41	(0.19, 0.88)	0.023	1.06	(0.41, 2.74)	0.909

OR = odds ratio; CI = confidence interval.

*Each model includes all predictors and controls for respondent’s role and clustering by division.

## Discussion

Our findings suggest that individuals in specialty settings are more likely than their primary care counterparts to believe that MU Stage 1 will divert attention from other patient care activities. We did not, however, find that specialty was associated with perceptions about management support or readiness for MU, which is unexpected given evidence that some of the Stage 1 MU objectives may be perceived as being primary-care centric [16]. This finding may reflect perceptions among UNC HCS specialists that MU is a priority for their clinics and that the clinics’ leadership will be able to support the change effort effectively.

Although we did not pre-specify a directional association focused on role in the practice setting, it is notable that physicians overall reported being less willing to change than advanced practice providers or nurses. Physicians may perceive the change burden falls primarily to them or that MU threatens their autonomy by placing constraints on their ability to structure the patient encounter. Faculty/attending physicians also were less likely to believe that their department would be able to address problems that arose during MU implementation as compared with residents/fellows. This finding may indicate skepticism among senior physicians resulting from previous experience with change efforts in the clinic. Interestingly, however, faculty/attending physicians were more willing to change their work practices for MU than were residents/fellows, which counters the belief that younger faculty are more willing to adapt to new technology. This finding may reflect the greater financial incentive from MU for faculty/attending physicians, as compared to residents/fellows, or a greater awareness of the financial implications of MU for the clinics. Another possible explanation for this finding is that residents are focused on learning and believe they are working at capacity during their training; therefore, they are unwilling to adapt to change.

Furthermore, we found evidence that individuals’ perceptions about MU appropriateness and management support are associated with their willingness to change practice behavior. However, we found no evidence that individuals’ perceptions about MU appropriateness and management support were associated with their reported ability to perform MU. These results suggest that leaders of health care organizations should pay attention to the perceptions that providers and clinical staff have about MU appropriateness and management support for MU. Change management efforts could focus on improving these perceptions if need be as it is feasible that doing so could improve willingness to change practices for MU. Approaches for improving perceptions might include communicating the rationale and potential benefits of MU (both financial and for patient care), developing organizational policies that support (e.g., incentivize) MU demonstration, and providing guidance (e.g., training, coaching) that reduces the burden of change effort on providers and clinical staff. For example, if providers in a particular setting do not believe MU is appropriate, implementation activities could incorporate success stories specific to the specialist’s patient population. Moreover, if members of a particular setting perceive that their management will not support the MU change effort or be able to solve MU implementation problems, more intensive coaching resources might be directed toward assisting managers, providers, and clinical staff in that setting.

Although our findings provide useful insights for practitioners, additional research is needed on readiness for change in the context of MU. Specifically, more work is needed to determine if perceptions about MU appropriateness and management support are not simply associated with willingness to change for MU but instead are causal factors. Furthermore, future research should identify predictors of ability to perform MU.

### Limitations

This study has some limitations. First, we did not perform reliability or validity assessments of the survey instrument used in the study. Furthermore, the measures of variables we analyzed are single survey items; therefore, there are no internal consistency statistics or other psychometric assessments to report. Second, all participants used the same EHR. Therefore, our results may not account for how perceptions of EHR usability are associated with perceived readiness for MU. Third, our study does not account for participants’ perceptions of the task demands (i.e., burden) and value of the MU change for them [9]. Future studies could include these variables to see if they contribute to the model beyond perceptions of appropriateness of MU and management support. Fourth, our findings suggest a correlation between some suggested predictors of readiness and self-reported measures of readiness; however, they do not indicate causation. For example, it is possible that individuals’ readiness for MU could influence their perceptions about departmental support for MU. Fifth, we did not adjust for multiple comparisons, but we report all associations that we tested. Had we applied a conservative Bonferroni correction for the 20 tests we conducted, we would have concluded that those with p < 0.0025 remained significant. Finally, results may be biased if there were systematic differences in perceptions among those choosing to participate in the survey versus not. Because completion of the survey was anonymous and voluntary and because recruitment was distributed widely across various health system listservs and EHR messages, we were not able to compare characteristics of respondents and non-respondents. However, all clinics surveyed had been using the same EHR for many years; therefore, it seems unlikely that respondents would represent only “early adopters” of health information technology.

## Conclusions

Efforts to demonstrate meaningful use are resource intensive for health care organizations. To minimize the risk of failure, it is necessary to identify implementation barriers that feasibly can be addressed. Our study suggests that physicians may be less likely than advanced practice providers and nursing staff to believe that their department will effectively solve MU implementation problems and are less willing to change their practice behavior for MU. Our findings are also consistent with theory that has suggested an individual’s perceptions about the appropriateness of MU and management support for MU demonstration will correspond with his/her willingness to change work practices for MU. Leaders of health care organizations should pay close attention to the perceptions that providers and clinical staff have about MU appropriateness and management support for MU, as well as willingness to change for MU, as these perceptions might be directly related to subsequent MU implementation.
